# Transcriptome Analysis of Genes Regulated by Cholesterol Loading in Two Strains of Mouse Macrophages Associates Lysosome Pathway and ER Stress Response with Atherosclerosis Susceptibility

**DOI:** 10.1371/journal.pone.0065003

**Published:** 2013-05-21

**Authors:** Stela Z. Berisha, Jeffrey Hsu, Peggy Robinet, Jonathan D. Smith

**Affiliations:** 1 Department of Cellular and Molecular Medicine, Lerner Research Institute, Cleveland Clinic, Cleveland, Ohio, United States of America; 2 Department of Molecular Medicine, Cleveland Clinic Lerner College of Medicine of Case Western Reserve University, Cleveland, Ohio, United States of America; University of Padova, Italy

## Abstract

Cholesterol loaded macrophages in the arterial intima are the earliest histological evidence of atherosclerosis. Studies of mouse models of atherosclerosis have shown that the strain background can have a significant effect on lesion development. We have previously shown that DBA/2 *ApoE^−/−^* mice have aortic root lesions 10-fold larger than AKR *ApoE^−/−^*mice. The current study analyzes the response to cholesterol loading of macrophages from these two strains. Macrophages from the atherosclerosis susceptible DBA/2 strain had significantly higher levels of total and esterified cholesterol compared to atherosclerosis resistant AKR macrophages, while free cholesterol levels were higher in AKR cells. Gene expression profiles were obtained and data were analyzed for strain, cholesterol loading, and strain-cholesterol loading interaction effects by a fitted linear model. Pathway and transcriptional motif enrichment were identified by gene set enrichment analysis. In addition to observed strain differences in basal gene expression, we identified many transcripts whose expression was significantly altered in response to cholesterol loading, including *P2ry13* and *P2ry14, Trib3*, *Hyal1*, *Vegfa*, *Ccr5*, *Ly6a*, and *Ifit3*. Eight pathways were significantly enriched in transcripts regulated by cholesterol loading, among which the lysosome and cytokine-cytokine receptor interaction pathways had the highest number of significantly regulated transcripts. Of the differentially regulated transcripts with a strain-cholesterol loading interaction effect, we identified three genes known to participate in the endoplasmic reticulum (ER) stress response, *Ddit3, Trib3* and *Atf4.* These three transcripts were highly up-regulated by cholesterol in AKR and either down-regulated or unchanged in loaded DBA/2 macrophages, thus associating a robust ER stress response with atherosclerosis resistance. We identified significant transcripts with strain, loading, or strain-loading interaction effect that reside within previously described quantitative trait loci as atherosclerosis modifier candidate genes. In conclusion, we characterized several strain and cholesterol induced differences that may lead to new insights into cellular cholesterol metabolism and atherosclerosis.

## Introduction

Atherosclerosis is a complex and progressive pathology of arteries that can be initiated by the accumulation and entrapment of lipoproteins in the extracellular matrix of the sub-endothelial intima layer [1], [2]. Monocytes migrate across the endothelial monolayer into the intimal layer where they differentiate into macrophages and take up modified low density lipoproteins by scavenger receptors [3], [4]. Once inside the cells modified LDL is degraded in lysosomes, depositing free cholesterol in the lysosomes that is transported to the endoplasmic reticulum for conversion into cholesterol esters and storage in lipid droplets [5], [6]. Cholesterol esters inside the lipid droplets can be hydrolyzed back into free cholesterol; and, recently, this hydrolysis has been shown to be mediated via autophagy delivering cholesterol esters to lysosomal acid lipase [7]. Free cholesterol can be exported out of the cells through passive diffusion, and/or protein mediated efflux via ATP-binding cassette transporters *ABCA1, ABCG1* or scavenger receptor *SR-BI* [8]–[12]. Macrophages cannot limit their uptake of cholesterol as the expression of scavenger receptors is not subject to feedback regulation by cellular cholesterol content [13], [14]. An imbalance between cholesterol influx and efflux leads to excessive accumulation of cholesterol in macrophages transforming them into cholesterol-engorged foam cells characteristic of fatty streaks [15]–[17].

The genetic background strain has been shown to effect atherosclerotic lesion area in hyperlipidemic *ApoE*−/− mice [18]. We have previously shown that chow-diet fed DBA/2 *ApoE^−/−^* mice have aortic root lesion areas that are 10-fold larger than those in AKR *ApoE^−/−^*mice at 16 weeks of age [19]. To gain insight on the effects of cholesterol loading on macrophage gene expression and its relation to atherosclerosis susceptibility, we incubated bone-marrow derived macrophages from atherosclerosis resistant AKR *ApoE^−/−^* and atherosclerosis susceptible DBA/2 *ApoE^−/−^* mice with acetylated LDL (AcLDL). We found that DBA/2 cells accumulate more total and esterified cholesterol, but less free cholesterol than the AKR macrophages. We investigated the effect of cholesterol on the macrophages transcriptome and identified transcripts that were differentially regulated by strain or cholesterol loading, and those transcripts in which there was a strain-loading interaction effect. Gene set enrichment analysis showed that some of the identified transcripts are involved in the lysosome pathway, and we found several transcripts that play a role in the endoplasmic reticulum stress response. We hypothesized that some of these differentially regulated transcripts could be responsible for the observed difference in atherosclerosis susceptibility between the AKR and DBA/2 strains. We identified differentially expressed transcripts as possible atherosclerosis modifier candidate genes residing within atherosclerosis QTLs previously characterized in a strain intercross between these two strains [19].

## Materials and Methods

### Mice

Age matched AKR and DBA/2 *ApoE^−/−^* mice were maintained on chow diet and sacrificed at 16 weeks of age. Plasma total cholesterol levels at time of sacrifice were 372.8±47.78 mg/ml and 912.7±104.3 mg/ml for AKR *ApoE−/−* and DBA/2 *ApoE−/−* mice, respectively. Femurs were collected to isolate and culture bone marrow monocytes.

#### Ethics Statement

All experiments were performed in accordance with protocols approved by the Cleveland Clinic Institutional Animal Care and Use Committee.

### Total, free, and esterified cholesterol quantification

Bone marrow derived macrophages (BMM) from AKR and DBA/2 *ApoE^−/−^* mice were cultured in p100 cell culture dishes for 13 days with 15% L-cell conditioned media, a source of macrophage colony stimulating factor (M-CSF). We performed control studies to compare BMM cholesterol loading by 24 h incubation with 50 µg/ml AcLDL, copper oxidized LDL, or native LDL; and, we only observed robust cholesterol loading with AcLDL at this dose, justifying its use in our subsequent studies. The cells were loaded for 48 h with 50 µg/ml AcLDL to allow foam cell formation with unloaded cells used as controls. Lipids were extracted and total, free and esterified cholesterol levels were quantified and normalized to cell protein as previously described [20]. Quadruplicate samples were assayed in triplicate and significance was determined by ANOVA with Newman-Keuls posttest using GraphPad Prism software.

### Loading of macrophages with acetylated LDL for transcriptome profiling

Differentiated BMM in p100 dishes from AKR and DBA/2 *ApoE^−/−^* mice were incubated for 24 hours in the presence or absence of 50 (experiment 2) or 100 µg/ml (experiment 1) AcLDL in quadruplicate. Different AcLDL doses were used due to altered efficiency of cholesterol loading of independent AcLDL preparations. After the incubation, the media and cholesterol were aspirated off, cells were washed twice, scrapped in 1 ml ice-cold PBS and transferred to microcentrifuge tubes. Cell pellets were obtained by centrifugation in a microcentrifuge at 5,000 rpm for 2 minutes at RT and kept on ice for subsequent use.

### Isolation of total RNA from BMM cell pellets

Total RNA was isolated from cell pellets using the RNeasy Mini Kit (Qiagen, Valencia, CA), following the manufacturer's protocol. On-column digestion with RNase-free DNase (Qiagen, Valencia, CA) was performed to remove genomic DNA. DNase was removed in the subsequent washing steps. RNA integrity was tested by overnight incubation of 200–500 ng of total RNA at 37°C and observation of the 18S and 28S ribosomal RNA bands on a 1.2% agarose/ethidium bromide gel.

### Hybridization and detection of gene transcripts

An aliquot of total RNA for each sample (∼ 2 µg) was submitted to the Genomic Core at Lerner Research Institute. Total RNA was used as template to synthesize single stranded cDNA following the Illumina protocol. Single stranded cDNA is then converted into double stranded cDNA, purified and *in vitro* transcribed (in the presence of biotinylated UTP and CTP) to produce biotin-labeled cRNA. Purified labeled cRNA from experiment 1 and 2 samples were hybridized respectively to MouseRef–8_v1 and Ref-8_v2 microarray chips (Illumina) for 16 hours at 58°C, according to the manufacturer's instructions. Eight samples were profiled on one chip with either 24,613 or 25,697 transcript probes per sample. To reduce the chip-to-chip variability two control and two cholesterol-loaded samples from each strain were put on each microarray chip. After hybridization, the arrays were washed several times and stained with streptavidin-Cy3. After the staining, the arrays were washed and scanned on the Illumina BeadArray Reader using Illumina BeadScan image data acquisition software.

### Microarray data analysis

Expression data were quantile normalized and log2 transformed using the R-package *lumi* [21]. 1,342 unique probes that hybridized to transcript sequences containing a strain-specific polymorphism or an indel (insertion/deletion) were excluded from further gene expression-related analysis. Sequence variations between AKR and DBA/2 (SNPs and insertions-deletions) were obtained from sequencing data generated by the Mouse Genomes Project: http://www.sanger.ac.uk/resources/mouse/genomes/. Probes were also removed if no sample had a detection p-value less than 0.01. 9,316 and 11,919 probes remained for further analysis in experiments 1 and 2, respectively. To determine strain effects on the transcriptome, only the results of unloaded bone marrow macrophages were analyzed. Log2 fold change, unadjusted p-values, and false discovery rate adjusted p-values were determined separately for the two independent datasets with the *limma* package in R [22], [23] using a fitted linear model with the following equation: Y_i_ = β_1_Strain+β_2_Loading+β_3_Strain-Loading, with the strain and loading βs as additive covariates and the strain-loading interaction β as an interactive covariate.

Gene set enrichment analysis was performed for all expressed transcripts to identify possible pathways altered by strain, loading and strain-loading interactions. The *romer* function in the *limma* package was used to test for gene set enrichment analysis [24], [25] with the Molecular Signature Database from the Broad Institute as gene sets [26]. We present and discuss further only the pathways that were significantly enriched using the permutation test p-value threshold of 1.0E-04. The MIAME compliant microarray dataset from this study is available through the Gene Expression Omnibus server, accession number GSE38736.

### Real-Time quantitative PCR (qPCR)

To validate the findings of gene transcript measurements by microarray, we assessed the expression of selected gene transcripts by using qPCR approach. The expression levels of tribbles homolog 3 (*Trib3*), activating transcription factor 4 (*Atf4*), ceroid-lipofuscinosis neuronal 5 (*Cln5*), and ATP-binding cassette sub-family G member 1 (*Abcg1*) were quantified relative to the endogenous control gene, ubiquitin C (*Ubc*) using pre-designed TaqMan gene expression assays (Applied Biosystems). Mean fold changes for each sample were calculated as previously described [27].

### Western blot

Unloaded and cholesterol loaded BMM from AKR and DBA/2 were lysed with 0.5 mL of M-PER mammalian protein extraction reagent (Pierce Biotechnology). Cell lysates (45 µg protein/lane) were loaded, separated on a 4–15% gradient polyacrylamide gel and transferred to PVDF membranes by semi-dry electroblotting. After blocking with rapid blocking buffer (Amresco, Cat # M325) for 1 hour, the membrane was incubated overnight with rabbit antibody to TRIB3 (Proteintech, Cat # 13300) at 4°C. The membrane was washed and further incubated with HRP goat anti-rabbit IgG (Amresco, Cat # N791) secondary antibody for 1 hour. The membrane was then exposed to an enhanced chemiluminescent system, and bands were visualized by exposure to X-ray film. After stripping, GAPDH protein was visualized as loading control using goat antibody to GAPDH (Abcam, Cat # ab9483), followed by HRP rabbit anti-goat IgG as described above. TRIB3 band density was quantified using Total Lab TL120 software (Nonlinear Dynamics) and normalized to the respective GAPDH band density.

## Results and Discussion

### AKR and DBA/2 macrophages respond differently to cholesterol loading

**Figure 1:**
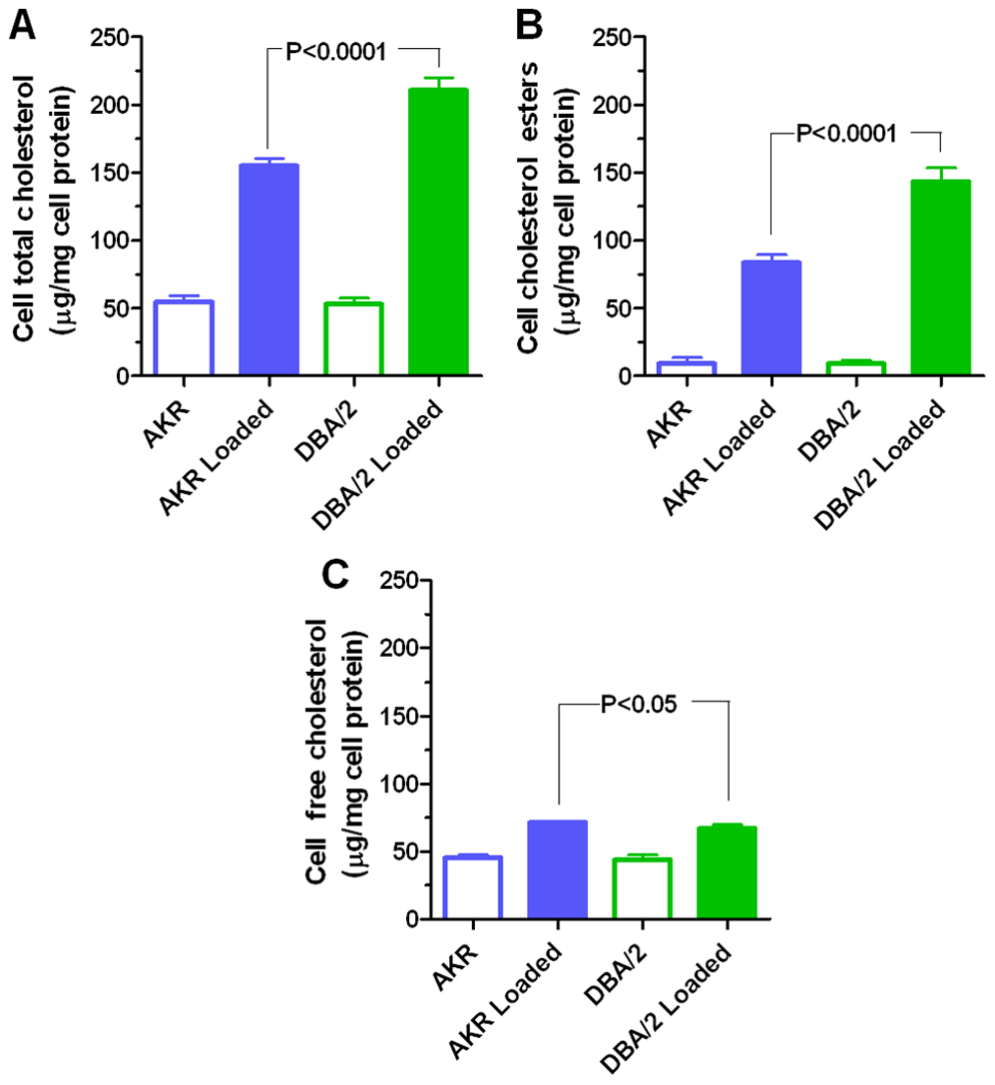
Total (A), esterified (B), and free (C) cholesterol mass in unloaded and AcLDL loaded AKR and DBA/2 *ApoE−/−* macrophages. All data are mean±SD, N = 4 per group using the average of triplicate assays per sample. P-values were calculated by ANOVA with Newman-Keuls posttest, showing only the strain differences after loading. There were no significant strain differences in unloaded cells.

Bone marrow derived macrophages of the two strains were incubated with 50 µg/ml AcLDL for 48 hours. We observed that macrophages from the atherosclerosis susceptible DBA/2 strain had significantly higher levels of total cholesterol (p-value<0.0001) and cholesterol esters (p-value <0.0001) compared to atherosclerosis resistant AKR macrophages (Figure 1A and 1B). In contrast, AcLDL-loaded DBA/2 macrophages had significantly lower levels of free cholesterol (p-value<0.05, Figure 1C). We have recently found that the strain difference in cholesterol ester metabolism is due to reduced cholesterol ester turnover in DBA/2 cells, which we attribute to decreased autophagic flux; and, autophagy is the primary mechanism responsible for the degradation of cholesterol esters in lipid droplets [28]. The current study focuses on the transcriptome differences between macrophages from the DBA/2 and AKR strains and the changes in response to cholesterol loading in these macrophages. Our objective was to identify possible genes and pathways that may play a role in the observed strain-specific differences in response to cholesterol loading and atherosclerosis.

### Hierarchical clustering

Bone marrow derived macrophages from mice of both strains were incubated with or without AcLDL for 1 day (n = 4 per group, total of 8 groups) in two independent experiments. Total RNA was applied to Illumina expression arrays and analyzed as described in Materials and Methods. Hierarchical clustering analysis was performed on all 32 samples (Figure S1). The two independent experiments were assayed on different versions of the Illumina array, using cells from different sexes with different batches of AcLDL, and were treated and analyzed in different years. Based on the observed clustering profile of these two groups, we decided not to pool the samples, and thus we analyzed the expression data from the two experiments separately for strain, loading and strain-loading interaction. Furthermore, we used the two studies to identify the overlapping group of genes and pathways that consistently respond to cholesterol loading in a strain-shared or strain-specific fashion.

### Strain differences on BMM transcriptome

#### Experiment 1 samples

3,059 transcripts were identified as differentially expressed between AKR and DBA/2 unloaded macrophages at a stringent false discovery rate (FDR) adjusted p-value<0.01 in experiment 1 samples. Table 1 shows the top 10 most significant differentially expressed transcripts, while the entire list is shown in Table S1. 229 differentially expressed transcripts were found with a 2- or higher fold change in expression between the two strains, of which 137 transcripts were expressed higher in DBA/2 and 92 transcripts were expressed higher in AKR macrophages.

**Table 1:** Top 10 differentially expressed transcripts between AKR and DBA/2 unloaded macrophages ranked by p-value.

	Gene Symbol	Log2Fold Change#	P-Value	Adjusted P-Value*	Description
Experiment 1	H2-D1	7.7	2.7E-23	2.5E-19	Histocompatibility 2, D region locus 1
	H2-Q6	5.6	1.1E-20	5.1E-17	Histocompatibility 2, Q region locus 6
	Gpr137b	4.2	1.5E-19	4.6E-16	G protein-coupled receptor 137B
	Ncf2	6.1	5.0E-19	1.2E-15	Neutrophil cytosolic factor 2
	Sfi1	3.7	7.0E-19	1.3E-15	Sfi1 homolog, spindle assembly associated (yeast)
	Psmb6	−3.7	9.4E-19	1.3E-15	Proteasome (prosome, macropain) subunit, beta type 6
	Ccl5	−4.5	9.2E-19	1.3E-15	Chemokine (C-C motif) ligand 5
	Baat1	3.2	1.8E-18	2.1E-15	BRCA1-associated ATM activator 1
	Gpnmb	−3.7	2.8E-18	2.9E-15	Glycoprotein (transmembrane) nmb
	Prcp	3.3	5.7E-18	5.3E-15	Prolylcarboxypeptidase (angiotensinase C)
Experiment 2	H2-K1	4.3	9.4E-14	1.1E-09	Histocompatibility 2, K region locus 1
	Pilrb1	5.2	5.2E-13	1.4E-09	Paired immunoglobin-like type 2 receptor beta 1
	H2-Q5	4.7	7.1E-13	1.4E-09	Histocompatibility 2, Q region locus 5
	H2-Q8	4.6	2.8E-12	1.4E-09	Histocompatibility 2, Q region locus 8
	Eif2s3y	4.4	3.9E-13	1.4E-09	Eukaryotic translation initiation factor 2, subunit 3, structural gene Y-linked
	Prcp	3.3	6.5E-13	1.4E-09	Prolylcarboxypeptidase (angiotensinase C)
	Lrrc57	3.6	5.0E-12	8.5E-09	Leucine rich repeat containing 57
	Ogfrl1	3.1	6.3E-12	9.3E-09	Opioid growth factor receptor-like 1
	Napsa	−3.1	7.2E-12	9.5E-09	Napsin A aspartic peptidase
	Baat1	3.3	8.1E-12	9.6E-09	BRCA1-associated ATM activator 1

# Positive log2 fold change, transcripts expressed higher in DBA/2; negative log2 fold change, transcript expressed higher in AKR macrophages.

* FDR adjusted p-value based upon permutation.

#### Experiment 2 samples

A similar analysis was performed on the experiment 2 samples and 1,703 transcripts were found to be differentially expressed between AKR and DBA/2 macrophages (FDR adjusted p-value<0.01; Table 1 top ten and Table S2 for all results). Of the 418 differentially expressed transcripts with a 2- or higher fold change, 220 transcripts were expressed higher in DBA/2 and 198 were expressed higher in AKR macrophages.

#### Combined results and discussion

522 differentially expressed transcripts overlapped among the 3,059 and 1,703 strain significant differences identified in the experiment 1 and 2, respectively (significance threshold set at 0.01 FDR, Table S3), making these transcript changes the ones that we are most confident of. Among the top ten differentially expressed transcripts, two and three were in the histocompatibility gene family in experiment 1 and 2, respectively; however, different members of genes in this family were observed in the two experiments. The only gene to make it to the top ten in both experiments was *Prcp*, encoding prolylcarboxypeptidase (also known as angiotensinase C), expressed significantly higher in DBA/2 macrophages. Transcripts encoding for transmembrane glycoprotein nmb *(Gpnmb)* and napsin A aspartic peptidase (*Napsa*) were expressed significantly higher in AKR macrophages.

#### Gene set enrichment analysis

To identify the common biological pathways most relevant to the genes that differ in expression between AKR and DBA/2 BMM, transcriptomes from both experiments were subjected to Gene Set Enrichment Analysis (GSEA) using KEGG pathways, and we report here only the pathways identified as significantly enriched in both. Strain effects on geneset enrichment were found for the hematopoietic cell lineage, chemokine signaling, toll like receptor signaling and aldosterone regulated sodium reabsorption pathways (permutation test p-value<0.0001, Table S4).

In conclusion, significant basal gene expression differences were observed between the two strains in both experiments, which need to be considered in the following analysis of gene expression changes in the AKR and DBA/2 macrophages in response to cholesterol loading.

### Cholesterol loading effect on BMM transcriptome

To identify the differentially regulated transcript in response to cholesterol loading, the expression data were fitted in a linear model with strain as an additive variable and strain-loading interaction as an interactive variable. This model identified transcripts whose expression was either up-regulated or down-regulated in one or both strains upon cholesterol loading.

#### Experiment 1 samples

3,758 transcripts were identified as differentially expressed in response to cholesterol loading in AKR and DBA/2 macrophages at an FDR adjusted p-value<0.01 (Table 2 top ten and Table S5 for all results). There were 261 differentially expressed transcripts with a 2-fold or higher change in expression upon cholesterol loading in one or both strains, of which 127 transcripts were up-regulated and 134 down-regulated.

**Table 2:** Top 10 significantly up-regulated or down-regulated transcripts in response to cholesterol loading ranked by p-value.

	Gene Symbol		Log2Fold Change#	P-Value	Adjusted P-Value*	Description	
Experiment 1	P2ry13		−2.8	7.8E-20	2.7E-16	Purinergic receptor P2Y, G-protein coupled 13	
	Clec4a3		−2.5	8.7E-20	2.7E-16	C-type lectin domain family 4, member A3	
	Npy		2.1	6.1E-20	2.7E-16	Neuropeptide Y	
	Ms4a6c		−2.3	4.6E-19	5.9E-16	Membrane-spanning 4-domains, subfamily A, member 6C	
	H2-Ab1		−2.3	3.7E-19	5.9E-16	Histocompatibility 2, class II antigen A, beta 1	
	Hyal1		2.3	5.7E-19	5.9E-16	Hyaluronoglucosaminidase 1	
	Trib3		2.6	5.1E-19	5.9E-16	Tribbles homolog 3 (Drosophila)	
	Ppap2b		2.7	4.9E-19	5.9E-16	Phosphatidic acid phosphatase type 2B	
	Chac1		2.9	3.6E-19	5.9E-16	ChaC, cation transport regulator-like 1 (E. coli)	
	Ly6a		−2.2	6.7E-19	6.2E-16	Lymphocyte antigen 6 complex, locus A	
Experiment 2	Ccr5	−2.9		3.8E-16	2.8E-12	Chemokine (C-C motif) receptor 5	
	Ccr5	−3.0		6.0E-16	2.8E-12	Chemokine (C-C motif) receptor 5	
	Ifit3	−3.3		7.1E-16	2.8E-12	Interferon-induced protein with tetratricopeptide repeats 3	
	Fcgr1	−2.0		1.2E-15	3.7E-12	Fc receptor, IgG, high affinity I	
	Ccr5	−2.9		1.8E-15	4.4E-12	Chemokine (C-C motif) receptor 5	
	P2ry14	−1.9		3.2E-15	6.4E-12	Purinergic receptor P2Y, G-protein coupled, 14	
	Trib3	3.7		4.8E-15	8.1E-12	Tribbles homolog 3 (Drosophila)	
	Ifit3	−3.2		6.4E-15	9.6E-12	Interferon-induced protein with tetratricopeptide repeats 3	
	Ly6a	−3.9		7.4E-15	9.8E-12	Lymphocyte antigen 6 complex, locus A	
	Vegfa	2.7		9.9E-15	1.2E-11	Vascular endothelial growth factor A	

# The fold change after cholesterol loading is represented as the β-coefficient for the fitted linear model.Positive log2 fold change, transcript expressed higher in response to cholesterol loading; negative log2 fold change, transcript expressed lower upon cholesterol loading.

* FDR adjusted p-value based upon permutation.

#### Experiment 2 samples

A similar number of significantly differentially expressed transcripts were found in macrophages in response to cholesterol loading (3,308 transcripts; FDR adjusted p-value<0.01; Table 2, Table S6). Of the 567 differentially expressed transcripts with a 2-fold or higher change, 236 transcripts were up-regulated upon cholesterol loading versus 331 that were down-regulated in one or both strains.

#### Combined results and discussion

The cholesterol loading effect on gene expression was largely reproducible in both experiments, despite microarray platform differences. There were 2,475 cholesterol regulated transcripts with identical probes on the two array platforms, and of these, 1140 transcripts were significantly regulated by cholesterol loading in both experiments (Table S7). The cholesterol-loading induced fold changes of these transcripts were also well correlated between the two experiments (R^2^ = 0.68, Figure S2).

Transcripts that were significantly up-regulated in response to cholesterol loading in both experiments include: tribbles homolog 3 (*Trib3*), hyaluronoglucosaminidase 1 (*Hyal1*), vascular endothelial growth factor A (*Vegfa*), etc. Transcripts that were significantly down-regulated in both experiments include: chemokine (C-C motif) receptor 5 (*Ccr5*), lymphocyte antigen 6 complex, locus A (*Ly6a*), interferon-induced protein with tetratricopeptide repeats 3 (*Ifit3*), etc. The related purinergic receptors *P2ry13* and *P2ry14* were also down regulated in both experiments, with *P2ry13* identified as the most significantly altered transcript in response to cholesterol loading in experiment 1, while *P2ry14* was identified among the top 10 most significant transcripts in experiment 2. *P2ry14* encodes for a G-protein coupled receptor expressed in a subpopulation of bone-marrow hematopoietic stem cells [29]. *P2ry13* has been shown to play a role in hepatic uptake of holo-HDL particles, particularly in SR-BI deficient liver cells; and, *P2ry13* knockout mice are reported to have decreased reverse cholesterol transport [30]. However, whether *P2ry13* deficiency has an effect on plasma HDL levels in mice is controversial [30], [31]. The most significantly altered transcript in response to cholesterol loading in experiment 2 was chemokine (C-C motif) receptor 5 (*Ccr5*), which was down-regulated by this treatment. Emerging evidence from both human and mouse studies supports important role(s) played by the *Ccr5* receptor and its ligand *Ccl5* in atherogenesis, a detailed description of which is provided in a recent review by Jones *et al.* [32].

#### Gene set enrichment analysis

Eight pathways were significantly enriched in transcripts regulated by cholesterol loading in both experiments: lysosome, cytokine-cytokine receptor interaction, primary bile acid biosynthesis, allograft rejection, aminoacyl tRNA biosynthesis, autoimmune thyroid disease, hematopoietic cell lineage, and type I diabetes mellitus (permutation test p-value<0.0001, Table S4). We looked more carefully at the lysosome pathway because it had the highest number of genes involved and of the recent discovery that macrophage cholesterol ester is mobilized for efflux via autophagy, with the cholesterol ester hydrolyzed by lysosomal acid lipase [7]. The number of significant cholesterol regulated transcripts in this pathway was 43 and 45 in experiment 1 and 2, respectively (Table 3), with 25 in common in both experiments. GSEA allows both the few strongly differentially expressed transcripts and the many weakly differentially expressed transcripts to factor into the enrichment analysis. Thus, most of the lysosomal pathway genes in Table 3 are only modestly regulated by cholesterol loading. Nevertheless, many minor changes in gene expression in the same pathway may add up to a large overall effect in that pathway, in this instance lysosome function.

**Table 3:** Significantly regulated transcripts upon cholesterol loading involved in lysosome pathway, ranked by fold change (loaded/unloaded).

Expt. 1 Gene Symbol	AKR Log2 Fold Change#	DBA Log2 Fold Change*	Adjusted P-Value	Expt. 2 Gene Symbol	AKR Log2 Fold Change#	DBA Log2 Fold Change*	Adjusted P-Value
Hyal1	2.3	0.6	5.9E-16	Hyal1	1.3	1.1	2.8E-07
Igf2r	1.4	0.6	2.3E-12	Atp6v1h	0.8	0.7	1.0E-06
Ctsk	1.3	0.4	9.6E-12	Sort1	0.8	1.6	4.8E-05
Tcirg1	1.3	0.2	2.9E-12	Gla	0.7	0.0	5.9E-04
Ctsb	0.9	0.1	1.1E-08	Ctsl	0.7	0.3	5.0E-04
Gla	0.7	0.1	1.5E-06	Ctsz	0.7	0.3	9.3E-05
Naglu	0.7	0.1	2.8E-08	Igf2r	0.6	1.3	2.2E-04
Gga2	0.7	0.3	1.8E-09	Cltb	0.6	0.3	1.2E-04
Cd68	0.6	0.1	8.6E-05	Mcoln1	0.6	0.5	8.6E-05
Gnptab	0.6	0.1	2.0E-07	Slc11a1	0.6	0.5	1.2E-05
Slc17a5	0.6	−0.1	1.5E-06	Dnase2a	0.6	0.5	8.2E-05
Cln5	0.6	0.3	6.8E-08	Tcirg1	0.6	0.6	2.0E-05
Atp6v0b	0.5	0.2	1.2E-06	Cln5	0.6	0.3	4.1E-05
Ctsz	0.5	0.2	6.8E-07	Ctsa	0.5	0.0	7.9E-03
Ctns	0.5	−0.1	6.9E-05	Ap1s1	0.5	0.3	1.9E-03
Ap3d1	0.5	−0.1	8.8E-06	Atp6v0a2	0.5	0.1	1.1E-04
Neu1	0.4	0.1	2.9E-04	Atp6v0d1	0.5	0.1	4.0E-04
Mcoln1	0.3	0.1	4.2E-04	Cd68	0.5	0.6	5.0E-03
Ctsd	0.3	0.1	9.3E-04	Gaa	0.4	0.3	5.8E-03
Abca2	0.3	0.1	5.9E-04	Gba	0.3	0.3	2.1E-03
Manba	0.3	0.1	3.2E-03	Psap	0.3	0.4	2.3E-03
Dnase2a	0.3	0.0	1.4E-03	Glb1	−0.3	0.1	3.4E-03
Ap1s1	0.3	0.4	1.1E-03	Lgmn	−0.4	−0.2	2.7E-03
Atp6v0a1	0.2	0.2	5.2E-03	Man2b1	−0.4	0.3	4.6E-04
Psap	0.2	0.0	5.1E-03	Ap4m1	−0.4	−0.3	5.0E-03
Acp2	−0.3	−0.1	2.1E-03	Pla2g15	−0.4	−0.2	2.5E-04
Ap4m1	−0.3	0.1	1.4E-03	Lamp2	−0.4	−0.7	7.8E-04
Ap1b1	−0.3	−0.2	2.2E-03	Ap3m2	−0.4	−0.3	4.1E-04
Ap3m2	−0.3	0.2	2.1E-03	Arsb	−0.4	0.1	6.8E-03
Ppt2	−0.3	0.0	1.0E-04	Smpd1	−0.5	−0.5	4.4E-03
Ctsh	−0.4	−0.1	2.2E-05	Acp2	−0.5	−1.0	2.5E-04
Sort1	−0.4	−0.3	5.6E-04	Clta	−0.5	−0.1	1.3E-05
Ap4s1	−0.4	0.0	1.5E-05	Ctss	−0.5	0.1	2.7E-03
Asah1	−0.5	−0.9	1.8E-03	Ctsf	−0.5	−0.2	4.6E-03
Clta	−0.5	0.3	1.6E-03	Manba	−0.6	0.1	3.5E-05
Lamp2	−0.5	0.0	4.6E-06	Sgsh	−0.6	0.4	3.7E-03
Slc11a1	−0.5	−0.2	1.6E-06	Gusb	−0.6	−0.2	2.3E-04
Ap1s2	−0.5	0.1	2.6E-06	Ppt1	−0.7	−0.5	4.1E-05
Napsa	−0.5	−0.1	3.1E-07	Ctsc	−0.7	−1.4	1.2E-04
Ctse	−0.7	−0.4	7.7E-08	Hgsnat	−0.8	−0.4	5.4E-05
Ppt1	−0.7	−0.3	1.4E-08	Ids	−0.8	−0.5	6.7E-07
Laptm4a	−0.8	−0.3	1.1E-08	Ctse	−0.8	−0.8	4.0E-07
Ctsc	−1.2	−0.1	8.9E-13	Asah1	−0.9	−1.1	6.9E-06
				Napsa	−0.9	−0.1	8.0E-06
				Ctsc	−1.2	−1.6	1.7E-08
				Ap1b1	−1.3	−0.3	9.9E-10

The 25 overlapping genes between the two experiments are shown in bold.

# Calculated as log2 of AKR loaded/AKR unloaded average expression, positive numbers higher in loaded, negative numbers higher in loaded.

* Calculated as log2 of DBA loaded/DBA unloaded average expression

Most of the lysosome pathway genes regulated by cholesterol showed a larger fold change in macrophages from the atherosclerosis resistant AKR than the susceptible DBA/2 strain, and a systematic analysis of the strain-cholesterol loading interaction is provided below. These experimental-validated regulated transcripts include the following lysosomal acid hydrolases: 1) proteases represented by cathepsins (*CtsC, CtsE* and *CtsZ*); 2) the peptidase napsin (*Napsa*); 3) glycosidases (*Hyal1* and *Gla*); 4) palmitoyl-protein thioesterase (*Ppt1*); 5) phosphatase (*Acp2*); and 6) ceramidase (*Asah1*). Point mutations in the *ASAH1* gene leads to the lysosomal storage disorder Farber disease; and, *Asah1+/−* mice develop an advanced lipid storage disease in many organs, most prominently in liver [33]. The conserved set of cholesterol regulated genes also includes: 1) several major and minor lysosomal membrane proteins (*Lamp2, Cd68* and *Cln5*); 2) adapter-related protein complex subunits beta, mu and sigma (*Ap3d1, Ap3m2* and *Ap1s1*) that are involved in the transport between lysosomes and Golgi; 3) sortilin 1 (*Sort1*); and 4) mucolipin1 *(Mcoln1)*. Of these non-hydrolytic lysosomal pathway genes, two stood out as potentially relevant to atherosclerosis *Mcoln1*and *Sort1*. *Mcoln1* encodes for a protein that co-localizes with endocytosed material that accumulates in lysosomes, and it plays a role in the exit of lipids from the lysosome and the trafficking of MHCII to the plasma membrane [34]. In addition, *Mcoln1*-deficient neurons have defective autophagic flux leading to increased levels of LC3-II and P62 [35]. *Sort1* is a genome wide association study (GWAS) hit for LDL-cholesterol and coronary artery disease, and has been shown to play a role in hepatic apoB lipoprotein secretion and LDL uptake [36].

#### SREBP and LXR motifs in cholesterol regulated transcripts

Since GSEA for sequence motifs was not very informative for the cholesterol loading datasets (data not shown); we examined motifs for two well known sterol regulated transcription factors. The classical example of cholesterol regulation of gene expression involves the down regulation of genes containing the sterol responsive element (SRE). This regulation is mediated by sterol control of sterol regulatory element binding protein (SREBP) processing in the ER and Golgi, such that high sterols repress its processing and low sterols permit its processing into a positively acting transcription factor [37]. Thus, we searched for the V$SREBP1_02 motif (KATCACCCCAC target sequence) motif within 2 kb of the transcription start site among all expressed transcripts in the two experiments. We identified 24 expressed transcripts associated with the V$SREBP1_02 motif in the first experiment. 11 transcripts (46%) were significantly regulated by cholesterol loading with 6 up regulated and 5 down regulated (Table S8). In the second experiment, we identified 35 expressed transcripts associated with the V$SREBP1_02 motif, 12 of which (34%) were significantly regulated by cholesterol loading with 4 up regulated and 8 down regulated. Seven replicated transcripts with this motif met our criteria for significant regulation by cholesterol loading in both experiments, two up and five down regulated (Table S8); however, the overlap between the two sets of cholesterol regulated SERPB1-associated transcripts was not significantly different compared to the overlap of all cholesterol regulated genes (one-tailed Fisher's exact test, p = 0.15). These seven replicated transcripts are not directly involved in cholesterol biosynthesis. A similar finding was reported in mouse macrophages deficient for SREBP1a, in which the most highly regulated transcripts did not include those coding for classical cholesterol biosynthesis genes [38]. Overall, we were surprised that several of the SREBP motif-containing genes were induced upon loading, a state where SREPB is expected to be low and the SRE containing genes are repressed. However, there are now well known examples of SREBP acting as a transcriptional repressor, such as SREBP repression of IRS2 transcription in liver [39].

**Figure 2:**
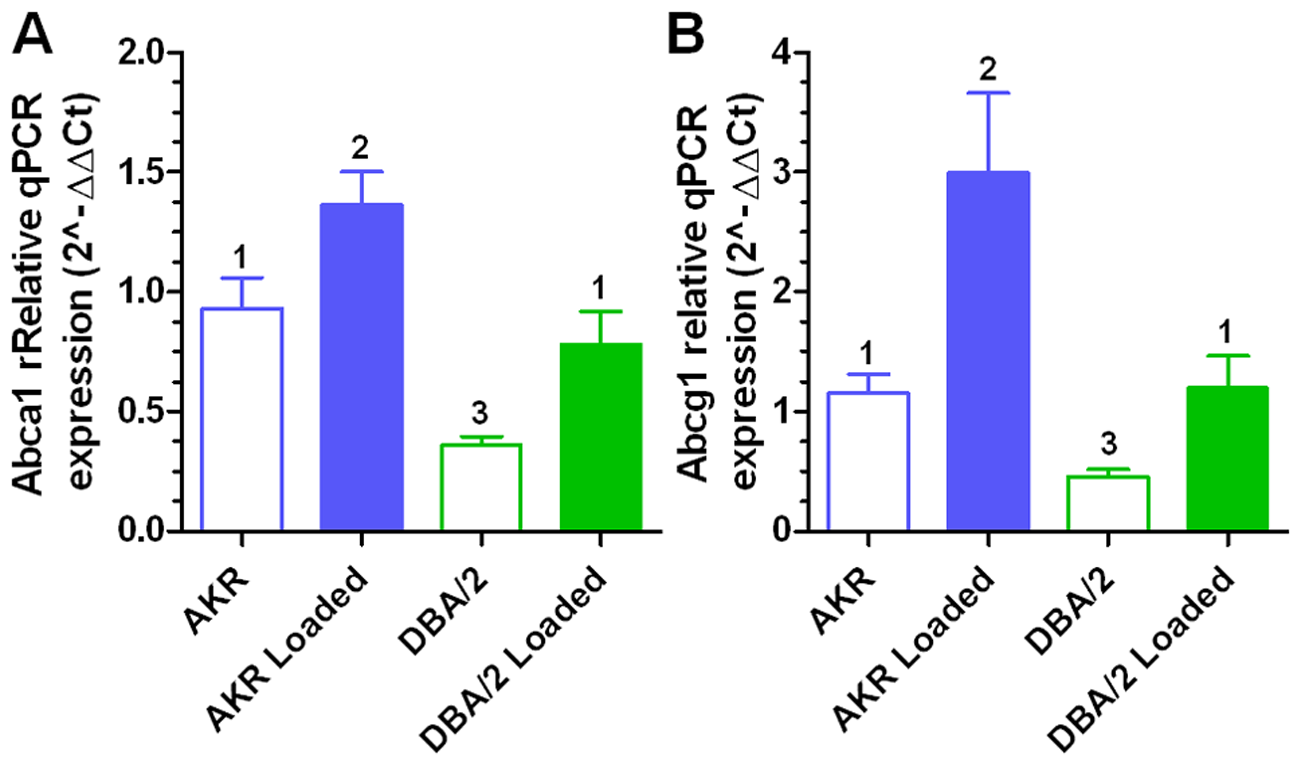
Gene expression and validation of microarray data by quantitative real-time PCR in experiment 1 and 2 macrophages. *Abca1* (A) and *Abcg1* (B) expression was calculated relative to ubiquitin C (*Ubc*) gene expression, an endogenous control whose expression remained unchanged under the conditions of experiment. Values are expressed as mean±SD (N = 4). Different numbers above bars show p<0.001 (A) or p<0.05 (B) by Newman-Keuls ANOVA posttest, while similar numbers above bars show no significant differences.

The oxysterol activated transcription factor LXR heterodimerizes with RXR and binds to genes harboring LXR responsive elements, often leading to sterol mediated up-regulated gene expression, as demonstrated for *Abca1*, *Abcg1*, and *apoE* [40]. We searched for the LXR DR4 motif TGACCGNNAGTRACCC within 2 kb of the start site of expressed transcripts. We identified 32 expressed transcripts associated with the LXR DR4 motif in the first experiment. 17 transcripts (53%) were significantly regulated by cholesterol loading with 6 up regulated and 11 down regulated (Table S9). In the second experiment, we identified 31 expressed transcripts associated with the LXR DR4 motif, of which 11 transcripts (55%) were significantly regulated by cholesterol loading with 7 up regulated and 4 down regulated. Six transcripts with this motif met our criteria for significant regulation by cholesterol loading in both experiments, two up and four down regulated (Table S9); however, the overlap between the two sets of cholesterol regulated LXR-associated transcripts was not significantly different compared to the overlap of all cholesterol regulated genes (one-tailed Fisher's exact test, p = 0.45). The two replicated up-regulated genes were *Abcg1* and *Stac2*. *Abcg1* is a well-known LXR target gene, but we did not identify *Abca1* another well-known target, and a*poE* is not expressed in our apoE-deficient macrophages. The expression of *Abca1* on our microarrays was very low, possibly due to poor probe design, so we quantified its expression in the experiment 1 samples by qPCR, and determined that it is indeed up-regulated by cholesterol loading in both strains (Figure 2). In a combined ChIP and gene expression study in human THP1 macrophages, LXR agonist treatment was found to lead to widespread up and down regulation of genes adjacent to confirmed LXR binding sites [41], confirming that LXR may act as either a transcriptional activator or repressor.

### Cholesterol loading–strain interaction effect on BMM transcriptome

A fitted linear model using strain and loading as additive covariates was used to identify the transcripts with a significant cholesterol loading-strain interaction effect. This interactive effect identifies transcripts that have different directions or degrees of cholesterol regulation between the two strains, for example, a transcript that is up regulated in AKR and down regulated in DBA/2, or a transcript that is highly up regulated in AKR but only moderately up regulated in DBA/2 and vice-versa.

#### Experiment 1 samples

**Figure 3:**
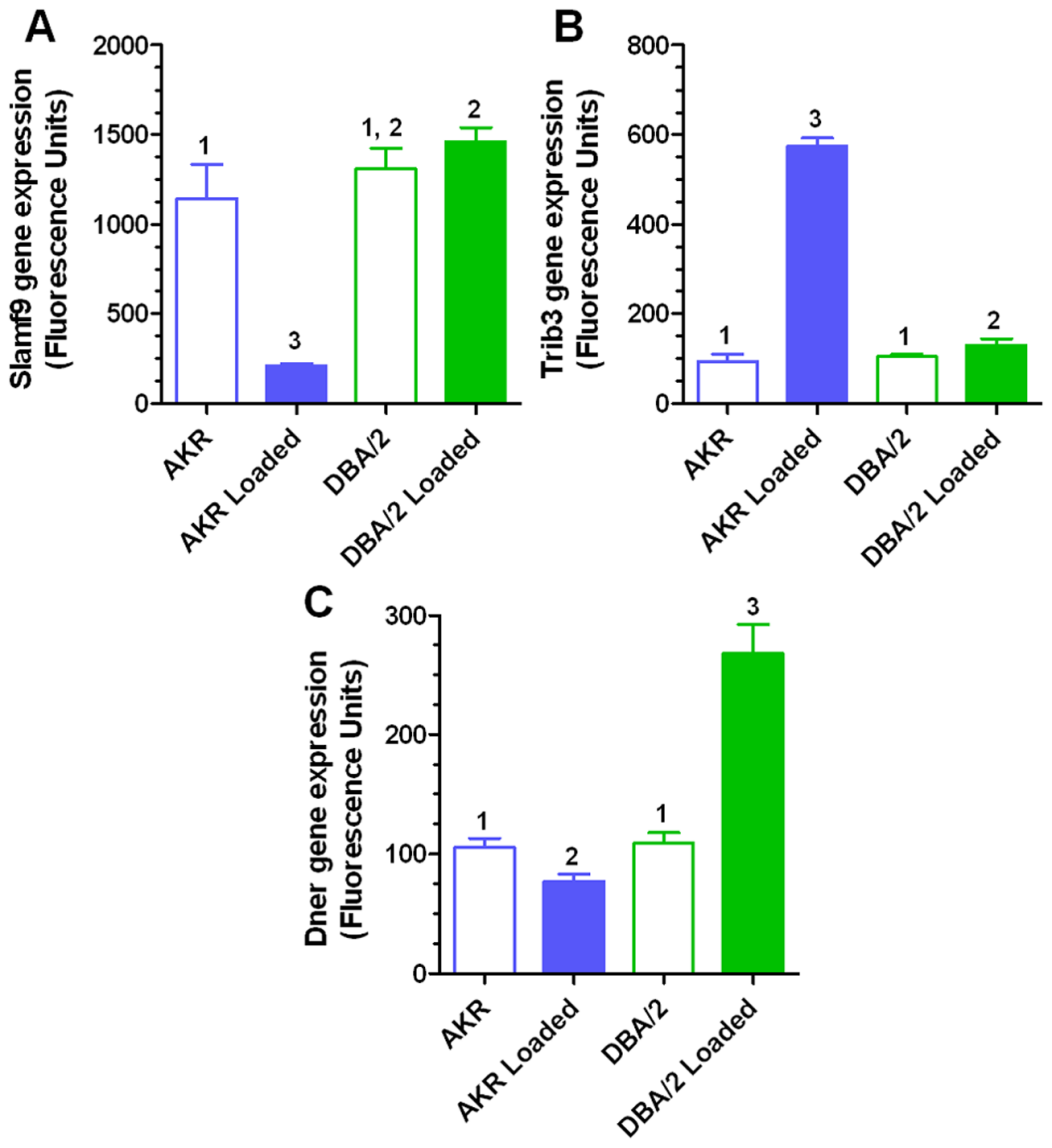
Examples of transcripts with strain-loading interaction effect in experiment 1 samples. Slamf9 (A), Trib3 (B), and Dner (C) expression in unloaded and loaded macrophages. Values are expressed as mean±SD (N = 4). Different numbers above bars show p<0.01 (A), p<0.05 (B, C) by Newman-Keuls ANOVA posttest, while similar numbers above bars show no significant differences.

1,929 probes were identified with a significant loading-strain interaction effect at an FDR adjusted p-value<0.01, with several transcripts independently identified by multiple probes (Table S10). The top 10 most significant transcripts for strain-loading interaction effect were: *Slamf9, Chac1, Trib3, Vwf, Gja1, Tgfbi, Dok2, Gadd45a, Gdf15* and *Dner.* The response of three of these transcripts to cholesterol loading in both strains is shown in Figure 3, with *Slamf9* highly down-regulated by loading in AKR (−5.4 fold) and not significantly changed in DBA/2; *Trib3* highly up-regulated in AKR (6.1 fold) and moderately up-regulated in DBA/2 (1.2 fold); and *Dner* down-regulated in AKR (−2.5 fold) and up-regulated in DBA/2 (1.4 fold).

#### Experiment 2 samples

**Figure 4:**
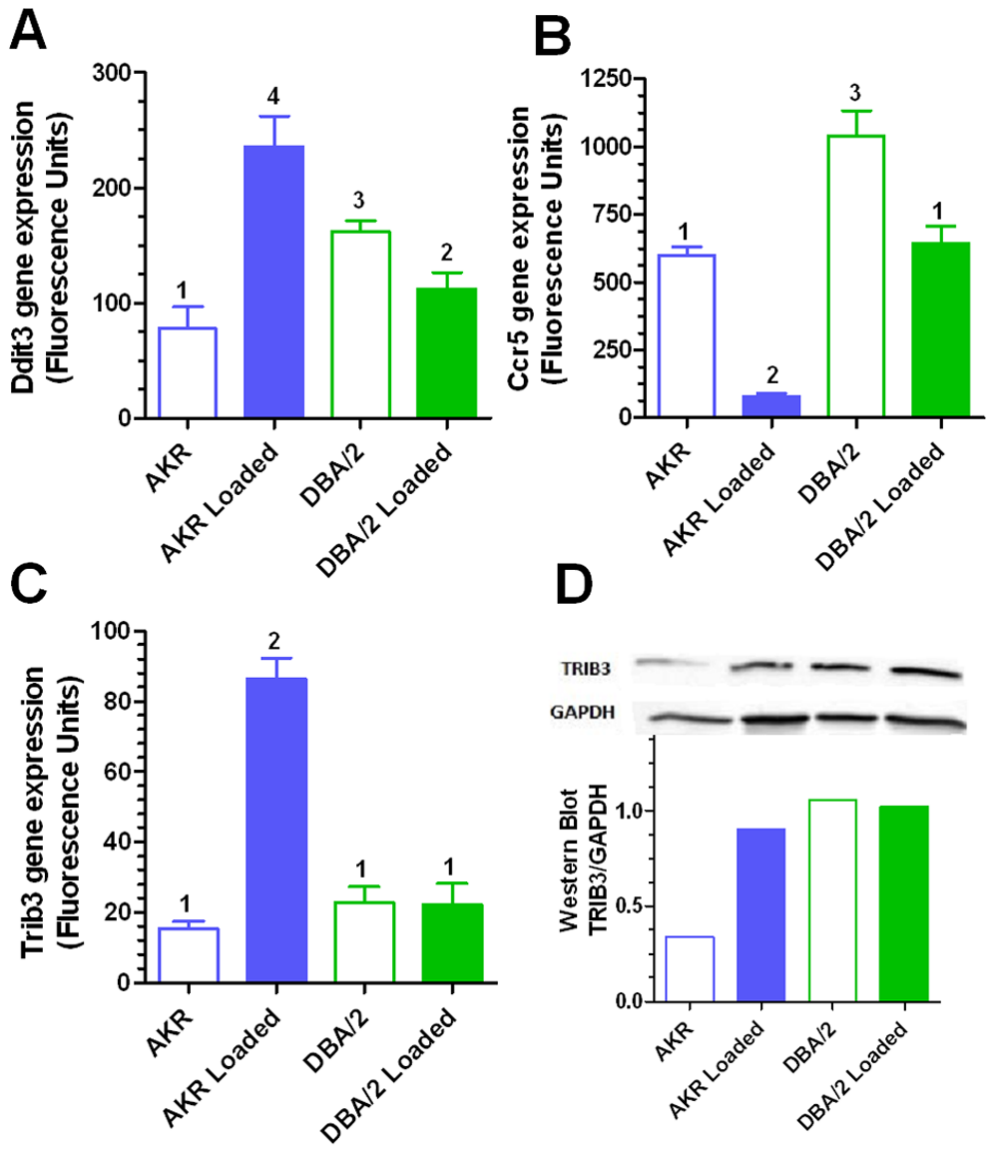
Examples of transcripts with strain-loading interaction effect in experiment 2 samples. Ddit3 (A), Ccr5 (B), and Trib3(C) expression in unloaded and loaded macrophages. Values are expressed as mean±SD (N = 4). Different numbers above bars show p<0.05 (A), p<0.001 (B), or p<0.001 (C) by Newman-Keuls ANOVA posttest, while similar numbers above bars show no significant differences. (D) Proteins isolated from unloaded and cholesterol loaded AKR and DBA/2 BMM were subjected to Western blot analysis, showing TRIB3 band density normalized to GAPDH.

965 probes were identified with a significant loading-strain interaction effect at an FDR adjusted p-value<0.01, with several transcripts independently identified by multiple probes (Table S11). The top 10 most significant probes for strain-loading interaction effect were: *Ddit3, Aqp9, Trib3, Nurp1, Cox6a2, Asns, Ccr5* (represented by three independent probes), and *Slc1a4.* The response of three of these transcripts to cholesterol loading in both strains is shown in Figure 4, with *Ddit3* up-regulated by loading in AKR (3.0 fold), and down-regulated in DBA/2 (−1.4 fold); *Trib3* up-regulated highly in AKR (5.6 fold) and unchanged in DBA/2; and *Ccr5* highly down-regulated in AKR (-7.6 fold) and moderately down-regulated in DBA/2 (−1.6 fold).

#### Combined results and discussion

There were 213 probes with highly significant strain-loading interactions that were conserved in both experiments (presented in Table S12). *Ddit3,* identified as the transcript with the most significant loading-strain interaction effect in the second experiment samples, was also observed with a significant strain loading effect in the first experiment. This gene encodes the DNA-damage inducible transcript 3 proteins, more widely known as CHOP (C/EBP homologous protein). CHOP is a transcription factor whose expression is up-regulated by endoplasmic reticulum (ER) stress response and the unfolded protein response (UPR), which under prolonged stress can trigger apoptosis [42], [43]. Two other genes known to participate in the ER stress pathway, *Trib3* and *Atf4* [44]–[46], were also among the list of genes with strain-loading interactions in both experiments with similar regulation as seen for *Ddit3*, highly up regulated by cholesterol loading in AKR, and either down regulated or unchanged in loaded DBA/2 cells. Tabas and colleagues have previously shown that free cholesterol loading of macrophages induces ER stress including CHOP production [47], [48]. Thus, our finding of increased expression of ER stress related genes in AKR, the strain with increased free cholesterol loading (Figure 1), fits well with the effects of free cholesterol on ER stress described by Tabas [47], [48].

ER stress, CHOP, and *Trib3* have previously been implicated in atherosclerosis. In *ApoE−/−* mice fed a western-type diet to induce advanced lesions, CHOP deficiency led to 35% smaller aortic lesions and 50% less plaque necrosis when compared to controls, despite similar levels of plasma lipoproteins (49). Similar results were obtained on the Ldlr-deficient background [49]. Adenoviral knockdown of *Trib3* in *ApoE*-*Ldlr* double knockout also led to smaller aortic lesions [50]. Our finding of *increased* ER stress response gene expression in cholesterol loaded macrophages from the atherosclerosis resistant AKR strain appears to differ from the above findings where *decreased* ER stress response was associated with smaller lesions. There are several potential reasons that might explain this discrepancy. One explanation is that the atherosclerosis studies comparing AKR and DBA/2 strains were performed in chow fed mice at an early time point prior to lesion necrosis, and Tabas has postulated that lesion macrophage apoptosis in early stage atherosclerosis is associated with increased phagocytic clearance of apoptotic cells and diminished lesion progression. Another explanation may be that ER stress can also trigger autophagy, which can be protective against lesion progression [51]. Autophagy also plays a role in cholesterol ester catabolism by delivering foam cell lipid droplets to the lysosome where lysosomal acid lipase cleaves cholesterol esters into free cholesterol, which is the substrate for cholesterol efflux [7].

### Validation of data by quantitative Real-Time PCR (qPCR)

**Figure 5:**
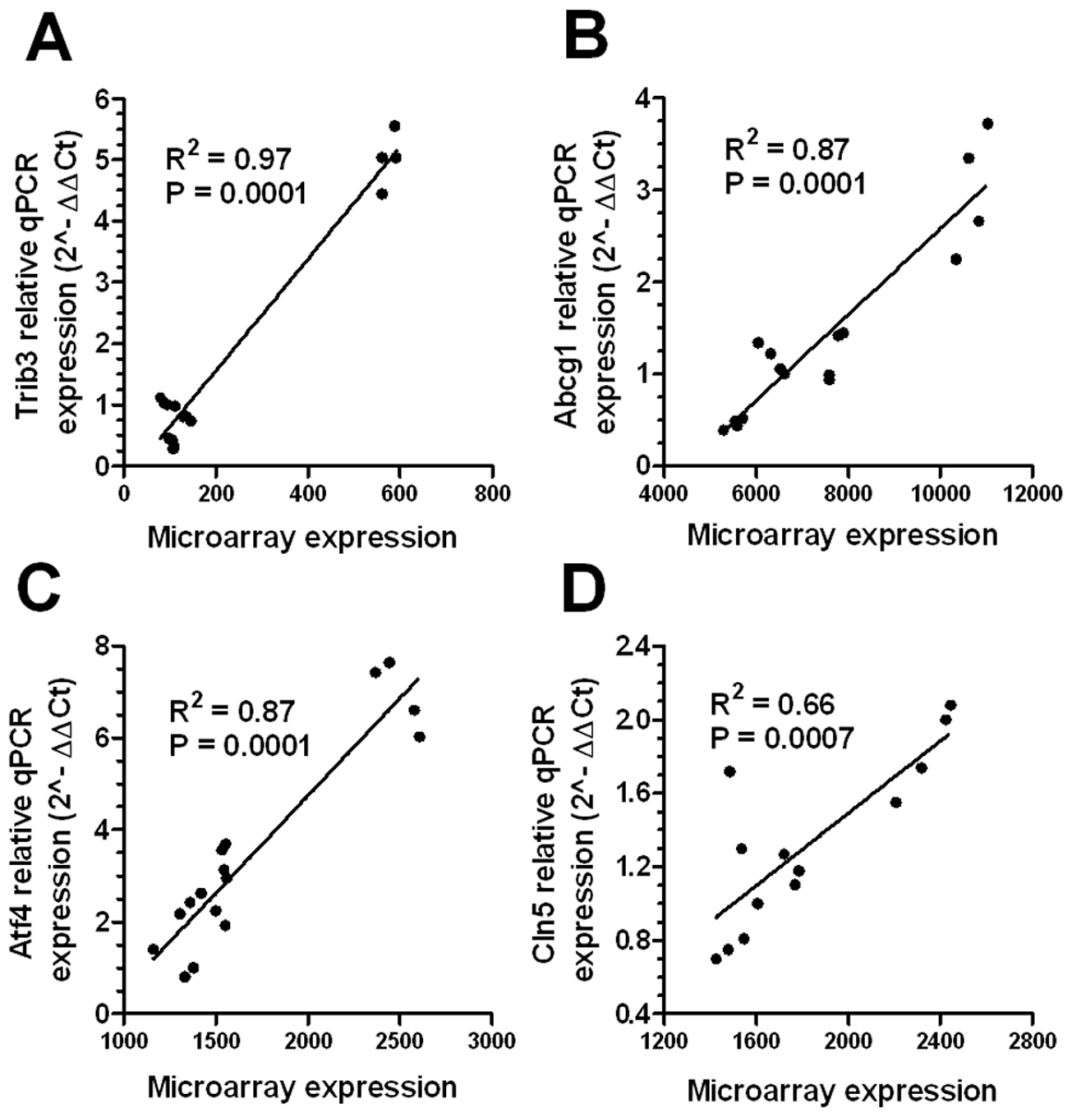
Validation of microarray expression data. Linear regression analysis of microarray expression and qPCR data for Trib3(A), Abcg1 (B), Atf4 (C) and Cln5 (D) performed in experiment 1 unloaded and loaded AKR and DBA/2 macrophages. Microarray data were not log2 transformed for this analysis.

To confirm the microarray data we performed quantitative real-time PCR for four selected transcripts: three highly regulated ones, *Trib3*, *Atf4* and *Cln5*, along with and LXR regulated transcript *Abcg1*. The expression levels for each transcript in the unloaded and cholesterol loaded samples from experiment 1 were quantified relative to the ubiquitin C (*Ubc*) control, which was found to be the least variable transcript in this study among the list of endogenous qPCR controls available from Applied Biosystems (∼3% coefficient of variation among the 16 samples in experiment 1). For each of these four transcripts, the expression levels quantified by qPCR were found to be consistent with the microarray data. Linear regression analysis revealed a highly significant positive correlation for each tested transcript (R^2^ values ranged from 0.66 to 0.97, Figure 5).

### Western Blot Analysis

We compared Trib3 mRNA levels (microarray) and protein levels (Western blot) in unloaded and cholesterol loaded AKR and DBA/2 BMM (Figure 4C and 4D). A large increase in Trib3 upon cholesterol loading in AKR cells was observed at both the mRNA and protein levels. In contrast, upon cholesterol loading in DBA/2 cells there was no appreciable change in Trib3 mRNA or protein. Thus, the strain-specific response to cholesterol was reproducibly observed at both the mRNA and protein levels. However, the basal levels of *Trib3* mRNA in the AKR and DBA/2 cells were similar, while Trib3 protein levels were much higher in DBA/2 cells. Differences observed between mRNA and protein levels are not uncommon in complex biological samples [52]. Previous studies comparing mRNA and protein abundance among different mouse strains have found limited correlations (mean R =  0.27), such that the mean association between protein and mRNA levels is only 7.3% comparing various strains [53]. Furthermore, this study showed that clinical traits among the various mouse strains were more strongly correlated with transcript levels than protein levels [53].

### Identification of Candidate Genes for *Ath22, Ath26 and Ath28* QTLs

Several studies have integrated genetics and genomics to identify plausible candidate genes for complex diseases. Previous studies in our lab have identified atherosclerosis QTLs from an AKRxDBA/2 *ApoE−/−* intercross [19], of which *Ath28* QTL on chromosome 2, *Ath22* on chromosome 15 and *Ath26* on chromosome 17 were recently validated in a second independent F_2_ cohort [54]. We searched the list of genes differentially expressed by strain, cholesterol loading, or the cholesterol loading-strain interaction, whose differential expression was conserved in both experiments. Table 4 shows this list of differentially regulated atherosclerosis modifier candidate genes that reside within these three QTL intervals each defined as 1-LOD score drop from the QTL peak. More candidates were identified within the *Ath26* QTL interval on chromosome 17, as this is the largest and most gene-dense QTL interval.

**Table 4:** Differentially expressed transcripts conserved in both experiments that reside within Ath28, Ath22 and Ath26 QTLs.

Chromosome 2# (*Ath28* QTL)	Chromosome 15$ (*Ath22* QTL)	Chromosome 17& (*Ath26* QTL)	
Slc13a3^a^	Capsl^a^	Tmem63ba, b	Srpk1^b^
Sdc4^a^	Myo10^a^	C2a, b	Gtpbp2b, c
Mmp9^a^	Oxct1^a^	Tmem8^a^	Nme4^b^
Rnf114^b^	Fam134b^a^	Ltba, b. c	Cfb^b^
Gnas^b^	Rai14^b^	Prss22^a^	Nrmb, c
Ctsz^b^	Il7r^b^	Rpl7l1^a^	Mapk14^b^
Gnas^b^	Ank^c^	Enpp4^a^	Angptl4^b^
Cebpbb, c		Itpr3^a^	Cdkn1a^b^
Rae1^b^		Tnfrsf21^a^	H2-T23
		Amdhd2^a^	B430306N03Rik^b^
		Fpr1a, b	Brd2^b^
		Cul7^a^	Cyp4f16^b^
		Fahd1^a^	Rrp1b^b^
		Emr1a, b, c	Tcf19b, c
		Mrpl14^a^	Aurka^b^
		Dusp1^a^	Pex6b, c
		Hagh^a^	Vars^b^
		Ubxn6^a^	Hmga1^b^
		Tubb5^a^	Trip10^b^
		Fpr2^b^	Klc4^b^
		Aif1b, c	Plcl2^b^
		Itpr3^b^	Cul7b, c
		Fgd2b, c	Slc35b2^b^
		Ebi3^b^	Mocs1^b^
		Tnfrsf12a^b^	Nfkbie^b^
		H2-Eb1^b^	Dpp9^b^
		9030025P20Rik^b^	Vegfab, c
		Ccnd3^b^	Atp6v0e^b^
		Uhrf1b, c	Gpr108^b^
		Cnpy3^b^	Alkbh7^b^
		Flywch2^b^	Chaf1a^c^
		D17H6S56E-5^b^	2410011O22Rik^c^
		Lemd2^b^	

a Transcripts significant for strain effect

b Transcripts significant for cholesterol loading effect

c Transcripts significant for cholesterol loading-strain interaction effect

# *Ath28* QTL confidence interval on chromosome 2 extends from 160 to 181Mb

$ *Ath22* QTL confidence interval on chromosome 15 extends from 3 to 33Mb

& *Ath26* QTL confidence interval on chromosome 17 extends from 12 to 65Mb

## Supporting Information

Figure S1 **Hierarchical clustering analysis of 32 samples included in the study.** Four replicates each for control and cholesterol loaded macrophages from AKR and DBA/2 strain, both independent experiments were included (loaded; unloaded samples).
(PDF)

Figure S2 **Conservation of cholesterol induced changes in macrophage gene expression in two independent experiments.** Linear regression analysis of log2 fold changes of the 1,140 overlapping transcripts between experiment 1and experiment 2 dataset that are significantly regulated by cholesterol loading in one or both strains. P-value of linear regression <0.0001
(PDF)

Table S1 **Differentially Expressed Transcripts between AKR and DBA in Unloaded BMM (experiment 1).**
(XLS)

Table S2 **Differentially Expressed Transcripts between AKR and DBA in Unloaded BMM (experiment 2).**
(XLS)

Table S3 **Differentially Expressed Transcripts between AKR and DBA/2 that Overlap between Experiment 1 and 2 Datasets in Unloaded BMM.**
(XLS)

Table S4 **Significantly represented biological pathways identified in experiment 1 and 2 datasets for strain, loading and strain-loading interaction effect by Gene Set Enrichment Analysis.**
(XLS)

Table S5 **Genes with a significant cholesterol loading effect in experiment 1 samples.** The fold change after cholesterol loading is represented as the beta-coefficient for the fitted linear model. Positive log2 fold change, transcript expressed higher in response to cholesterol loading; negative log2 fold change, transcript expressed lower upon cholesterol loading.
(XLS)

Table S6 **Genes with a significant cholesterol loading effect in experiment 2 samples.**
(XLS)

Table S7 **Regulated transcripts with a significant cholesterol loading effect that overlap between experiment 1 and 2 datasets.**
(XLS)

Table S8 **Significant transcripts for cholesterol loading effect, with promoter regions 2 Kb to +2 Kb from the start site of transcription containing the motif KATCACCCCAC, which matches annotation for SREBF1: sterol regulatory element binding transcription factor 1.**
(XLS)

Table S9 **Significant transcripts for cholesterol loading effect, with promoter regions 2 Kb to +2 Kb from the start site of transcription containing the motif TGACCGNNAGTRACCC, which matches annotation for NR1H3: nuclear receptor subfamily 1, group H, member 3 (DR4).**
(XLS)

Table S10 **Genes with a significant strain-cholesterol loading interaction effect in experiment 1.** Strength of loading interaction effect on fitted model (with direction relative to expected DBA/2 response to cholesterol loading).
(XLS)

Table S11 **Genes with a significant strain-cholesterol loading interaction effect in experiment 2.**
(XLS)

Table S12 **Regulated transcripts with a significant cholesterol loading-strain interaction effect that overlap between experiment 1 and 2 datasets.**
(XLS)

## References

[1] Libby P, Ridker PM, Hansson GK (2011) Progress and challenges in translating the biology of atherosclerosis. Nature 473: 317–25. 21593864 10.1038/nature10146

[2] Glass CK, Witztum JL (2001) Atherosclerosis: the road ahead. Cell 104: 503–16. 11239408 10.1016/s0092-8674(01)00238-0

[3] Moore KJ, Freeman MW (2006) Scavenger receptors in atherosclerosis: Beyond lipid uptake. Arterioscler Thromb Vasc Biol 26: 1702–11. 16728653 10.1161/01.ATV.0000229218.97976.43

[4] Greaves DR, Gordon S (2009) The macrophage scavenger receptor at 30 years of age: Current knowledge and future challenges. J Lipid Res 50 Suppl: S282–6 10.1194/jlr.R800066-JLR200 PMC2674758 19074372

[5] Soccio RE, Breslow JL (2004) Intracellular cholesterol transport. Arterioscler Thromb Vasc Biol 24: 1150–60. 15130918 10.1161/01.ATV.0000131264.66417.d5

[6] Tiwari RL, Singh V, Barthwal MK (2008) Macrophages: An elusive yet emerging therapeutic target of atherosclerosis. Med Res Rev 28: 483–544. 18000963 10.1002/med.20118

[7] Ouimet M, Franklin V, Mak E, Liao X, Tabas I, et al (2011) Autophagy regulates cholesterol efflux from macrophage foam cells via lysosomal acid lipase. Cell Metab 13: 655–67. 21641547 10.1016/j.cmet.2011.03.023 PMC3257518

[8] Alwaili K, Awan Z, Alshahrani A, Genest J (2010) High-density lipoproteins and cardiovascular disease: 2010 update. Expert Rev Cardiovasc Ther 8: 413–23. 20222819 10.1586/erc.10.4

[9] Moreno PR, Sanz J, Fuster V (2009) Promoting mechanisms of vascular health: Circulating progenitor cells, angiogenesis, and reverse cholesterol transport. J Am Coll Cardiol 53: 2315–23. 19539140 10.1016/j.jacc.2009.02.057

[10] Wang X, Rader DJ (2007) Molecular regulation of macrophage reverse cholesterol transport. Curr Opin Cardiol 22: 368–72. 17556891 10.1097/HCO.0b013e3281ec5113

[11] Yvan-Charvet L, Wang N, Tall AR (2010) Role of HDL, ABCA1, and ABCG1 transporters in cholesterol efflux and immune responses. Arterioscler Thromb Vasc Biol 30: 139–43. 19797709 10.1161/ATVBAHA.108.179283 PMC2812788

[12] Marcel YL, Ouimet M, Wang MD (2008) Regulation of cholesterol efflux from macrophages. Curr Opin Lipidol 19: 455–61. 18769226 10.1097/MOL.0b013e32830f4a1d

[13] Pennings M, Meurs I, Ye D, Out R, Hoekstra M, et al (2006) Regulation of cholesterol homeostasis in macrophages and consequences for atherosclerotic lesion development. FEBS Lett 580: 5588–96. 16935283 10.1016/j.febslet.2006.08.022

[14] Vainio S, Ikonen E (2003) Macrophage cholesterol transport: A critical player in foam cell formation. Ann Med 35: 146–55. 12822736 10.1080/07853890310008198

[15] Shashkin P, Dragulev B, Ley K (2005) Macrophage differentiation to foam cells. Curr Pharm Des 11: 3061–72. 16178764 10.2174/1381612054865064

[16] Lusis AJ (2000) Atherosclerosis. Nature 407: 233–41. 11001066 10.1038/35025203 PMC2826222

[17] Lusis AJ, Mar R, Pajukanta P (2004) Genetics of atherosclerosis. Annu Rev Genomics Hum Genet 5: 189–218. 15485348 10.1146/annurev.genom.5.061903.175930

[18] Smith JD, James D, Dansky HM, Wittkowski KM, Moore KJ, et al (2003) In silico quantitative trait locus map for atherosclerosis susceptibility in apolipoprotein E-deficient mice. Arterioscler Thromb Vasc Biol 23: 117–22. 12524234 10.1161/01.atv.0000047461.18902.80

[19] Smith JD, Bhasin JM, Baglione J, Settle M, Xu Y, et al (2006) Atherosclerosis susceptibility loci identified from a strain intercross of apolipoprotein E-deficient mice via a high-density genome scan. Arterioscler Thromb Vasc Biol 26: 597–603. 16373612 10.1161/01.ATV.0000201044.33220.5c

[20] Robinet P, Wang Z, Hazen SL, Smith JD (2010) A simple and sensitive enzymatic method for cholesterol quantification in macrophages and foam cells. J Lipid Res 51: 3364–9. 20688754 10.1194/jlr.D007336 PMC2952578

[21] Du P, Kibbe WA, Lin SM (2008) Lumi: A pipeline for processing Illumina microarray. Bioinformatics 24: 1547–8. 18467348 10.1093/bioinformatics/btn224

[22] Smyth GK (2004) Linear models and empirical bayes methods for assessing differential expression in microarray experiments. Stat Appl Genet Mol Biol 3: Article3. 16646809 10.2202/1544-6115.1027

[23] Gentleman R (2005) Bioinformatics and computational biology solutions using R and bioconductor. New York: Springer.

[24] Dorum G, Snipen L, Solheim M, Saebo S (2009) Rotation testing in gene set enrichment analysis for small direct comparison experiments. Stat Appl Genet Mol Biol 8: Article34. 19645689 10.2202/1544-6115.1418

[25] Majewski IJ, Ritchie ME, Phipson B, Corbin J, Pakusch M, et al (2010) Opposing roles of polycomb repressive complexes in hematopoietic stem and progenitor cells. Blood 116: 731–9. 20445021 10.1182/blood-2009-12-260760

[26] Subramanian A, Tamayo P, Mootha VK, Mukherjee S, Ebert BL, et al (2005) Gene set enrichment analysis: A knowledge-based approach for interpreting genome-wide expression profiles. Proc Natl Acad Sci U S A 102: 15545–50. 16199517 10.1073/pnas.0506580102 PMC1239896

[27] Livak KJ, Schmittgen TD (2001) Analysis of relative gene expression data using real-time quantitative PCR and the 2(-delta delta C(T)) method. Methods 25: 402–8. 11846609 10.1006/meth.2001.1262

[28] Robinet P, Ritchey B, Smith JD (2013) Physiological difference in autophagic flux in macrophages from 2 mouse strains regulates cholesterol ester metabolism. Arterioscler Thromb Vasc Biol (In Press). 10.1161/ATVBAHA.112.301041 PMC3646371 23493286

[29] Lee BC, Cheng T, Adams GB, Attar EC, Miura N, et al (2003) P2Y-like receptor, GPR105 (P2Y14), identifies and mediates chemotaxis of bone-marrow hematopoietic stem cells. Genes Dev 17: 1592–604. 12842911 10.1101/gad.1071503 PMC196132

[30] Fabre AC, Malaval C, Ben Addi A, Verdier C, Pons V, et al (2010) P2Y13 receptor is critical for reverse cholesterol transport. Hepatology 52: 1477–83. 20830789 10.1002/hep.23897

[31] Blom D, Yamin TT, Champy MF, Selloum M, Bedu E, et al (2010) Altered lipoprotein metabolism in P2Y(13) knockout mice. Biochim Biophys Acta 1801: 1349–60. 20817122 10.1016/j.bbalip.2010.08.013

[32] Jones KL, Maguire JJ, Davenport AP (2011) Chemokine receptor CCR5: From AIDS to atherosclerosis. Br J Pharmacol 162: 1453–69. 21133894 10.1111/j.1476-5381.2010.01147.x PMC3057285

[33] Li CM, Park JH, Simonaro CM, He X, Gordon RE, et al (2002) Insertional mutagenesis of the mouse acid ceramidase gene leads to early embryonic lethality in homozygotes and progressive lipid storage disease in heterozygotes. Genomics 79: 218–24. 11829492 10.1006/geno.2002.6686

[34] Thompson EG, Schaheen L, Dang H, Fares H (2007) Lysosomal trafficking functions of mucolipin-1 in murine macrophages. BMC Cell Biol 8: 54. 18154673 10.1186/1471-2121-8-54 PMC2254603

[35] Curcio-Morelli C, Charles FA, Micsenyi MC, Cao Y, Venugopal B, et al (2010) Macroautophagy is defective in mucolipin-1-deficient mouse neurons. Neurobiol Dis 40: 370–7. 20600908 10.1016/j.nbd.2010.06.010 PMC4392647

[36] Strong A, Ding Q, Edmondson AC, Millar JS, Sachs KV, et al (2012) Hepatic sortilin regulates both apolipoprotein B secretion and LDL catabolism. J Clin Invest 122: 2807–16. 22751103 10.1172/JCI63563 PMC3408750

[37] Ye J, DeBose-Boyd RA (2011) Regulation of cholesterol and fatty acid synthesis. Cold Spring Harb Perspect Biol 3: 10. 10.1101/cshperspect.a004754 PMC3119913 21504873

[38] Im SS, Yousef L, Blaschitz C, Liu JZ, Edwards RA, et al (2011) Linking lipid metabolism to the innate immune response in macrophages through sterol regulatory element binding protein-1a. Cell Metab 13: 540–9. 21531336 10.1016/j.cmet.2011.04.001 PMC3090630

[39] Ide T, Shimano H, Yahagi N, Matsuzaka T, Nakakuki M, et al (2004) SREBPs suppress IRS-2-mediated insulin signaling in the liver. Nat Cell Biol 6: 351–7. 15048126 10.1038/ncb1111

[40] Zhao C, Dahlman-Wright K (2010) Liver X receptor in cholesterol metabolism. J Endocrinol 204: 233–40. 19837721 10.1677/JOE-09-0271

[41] Pehkonen P, Welter-Stahl L, Diwo J, Ryynanen J, Wienecke-Baldacchino A, et al (2012) Genome-wide landscape of liver X receptor chromatin binding and gene regulation in human macrophages. BMC Genomics 13: 50. 22292898 10.1186/1471-2164-13-50 PMC3295715

[42] Ron D, Walter P (2007) Signal integration in the endoplasmic reticulum unfolded protein response. Nat Rev Mol Cell Biol 8: 519–29. 17565364 10.1038/nrm2199

[43] Cazanave SC, Elmi NA, Akazawa Y, Bronk SF, Mott JL, et al (2010) CHOP and AP-1 cooperatively mediate PUMA expression during lipoapoptosis. Am J Physiol Gastrointest Liver Physiol 299: G236–43. 20430872 10.1152/ajpgi.00091.2010 PMC2904106

[44] Shang YY, Wang ZH, Zhang LP, Zhong M, Zhang Y, et al (2009) TRB3, upregulated by ox-LDL, mediates human monocyte-derived macrophage apoptosis. FEBS J 276: 2752–61. 19389115 10.1111/j.1742-4658.2009.06998.x

[45] Shang YY, Zhong M, Zhang LP, Guo ZX, Wang ZH, et al (2010) Tribble 3, a novel oxidized low-density lipoprotein-inducible gene, is induced via the activating transcription factor 4-C/EBP homologous protein pathway. Clin Exp Pharmacol Physiol 37: 51–5. 19566842 10.1111/j.1440-1681.2009.05229.x

[46] Prudente S, Sesti G, Pandolfi A, Andreozzi F, Consoli A, et al (2012) The mammalian tribbles homolog TRIB3, glucose homeostasis, and cardiovascular diseases. Endocr Rev 33: 526–46. 22577090 10.1210/er.2011-1042 PMC3410226

[47] Devries-Seimon T, Li Y, Yao PM, Stone E, Wang Y, et al (2005) Cholesterol-induced macrophage apoptosis requires ER stress pathways and engagement of the type A scavenger receptor. J Cell Biol 171: 61–73. 16203857 10.1083/jcb.200502078 PMC2171235

[48] Tabas I (2005) Consequences and therapeutic implications of macrophage apoptosis in atherosclerosis: The importance of lesion stage and phagocytic efficiency. Arterioscler Thromb Vasc Biol 25: 2255–64. 16141399 10.1161/01.ATV.0000184783.04864.9f

[49] Thorp E, Li G, Seimon TA, Kuriakose G, Ron D, et al (2009) Reduced apoptosis and plaque necrosis in advanced atherosclerotic lesions of apoe−/− and ldlr−/− mice lacking CHOP. Cell Metab 9: 474–81. 19416717 10.1016/j.cmet.2009.03.003 PMC2695925

[50] Wang ZH, Shang YY, Zhang S, Zhong M, Wang XP, et al (2012) Silence of TRIB3 suppresses atherosclerosis and stabilizes plaques in diabetic ApoE−/−/LDL receptor−/− mice. Diabetes 61: 463–73. 22275087 10.2337/db11-0518 PMC3266419

[51] Liao X, Sluimer JC, Wang Y, Subramanian M, Brown K, et al (2012) Macrophage autophagy plays a protective role in advanced atherosclerosis. Cell Metab 15: 545–53. 22445600 10.1016/j.cmet.2012.01.022 PMC3322248

[52] Maier T, Guell M, Serrano L (2009) Correlation of mRNA and protein in complex biological samples. FEBS Lett 583: 3966–73. 19850042 10.1016/j.febslet.2009.10.036

[53] Ghazalpour A, Bennett B, Petyuk VA, Orozco L, Hagopian R, et al (2011) Comparative analysis of proteome and transcriptome variation in mouse. PLoS Genet 7: e1001393. 21695224 10.1371/journal.pgen.1001393 PMC3111477

[54] Hsu J, Smith JD (2013) Genetic-Genomic replication to identify candidate mouse atherosclerosis modifier genes. Journal of the American Heart Association 2: e005421. 23525445 10.1161/JAHA.112.005421 PMC3603265

