# Supplementary material for: Transcriptome Analysis of Genes Regulated by Cholesterol Loading in Two Strains of Mouse Macrophages Associates Lysosome Pathway and ER Stress Response with Atherosclerosis Susceptibility
Source: PLoS One. 2013 May 21;8(5):e65003. doi: 10.1371/journal.pone.0065003 (PMC3660362; doi:10.1371/journal.pone.0065003)
Supplement: Figure S1 — Hierarchical clustering analysis of 32 samples included in the study. Four replicates each for control and cholesterol loaded macrophages from AKR and DBA/2 strain, both independent experiments were included (loaded; unloaded samples). (PDF) [file pone.0065003.s001.pdf]

Supplemental Figure S1

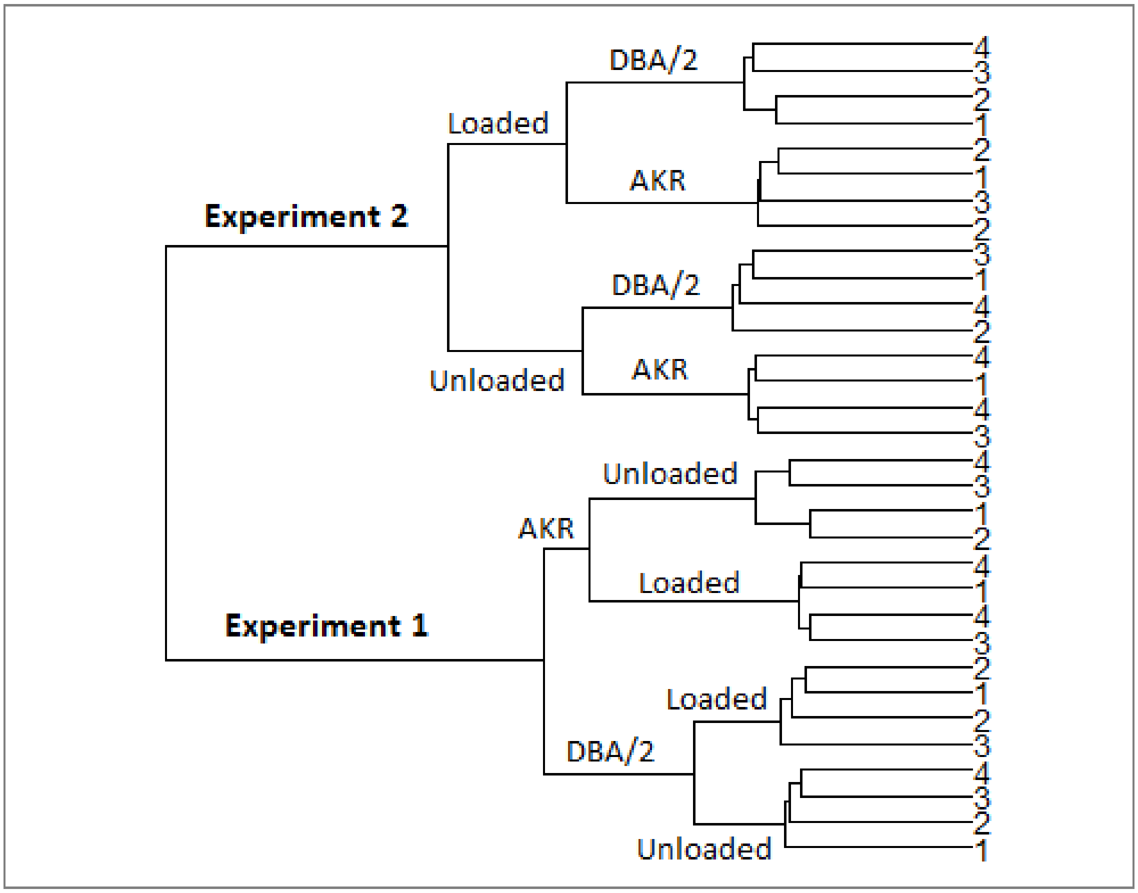

***Figure S1*: Hierarchical clustering analysis of 32 samples included in the study.** Four replicates each for control and cholesterol loaded macrophages from AKR and DBA/2 strain, both independent experiments were included (loaded; unloaded samples).
