# Supplementary material for: Transcriptome Analysis of Genes Regulated by Cholesterol Loading in Two Strains of Mouse Macrophages Associates Lysosome Pathway and ER Stress Response with Atherosclerosis Susceptibility
Source: PLoS One. 2013 May 21;8(5):e65003. doi: 10.1371/journal.pone.0065003 (PMC3660362; doi:10.1371/journal.pone.0065003)
Supplement: Figure S2 — Conservation of cholesterol induced changes in macrophage gene expression in two independent experiments. Linear regression analysis of log2 fold changes of the 1,140 overlapping transcripts between experiment 1and experiment 2 dataset that are significantly regulated by cholesterol loading in one or both strains. P-value of linear regression <0.0001 (PDF) [file pone.0065003.s002.pdf]

Supplemental Figure S2

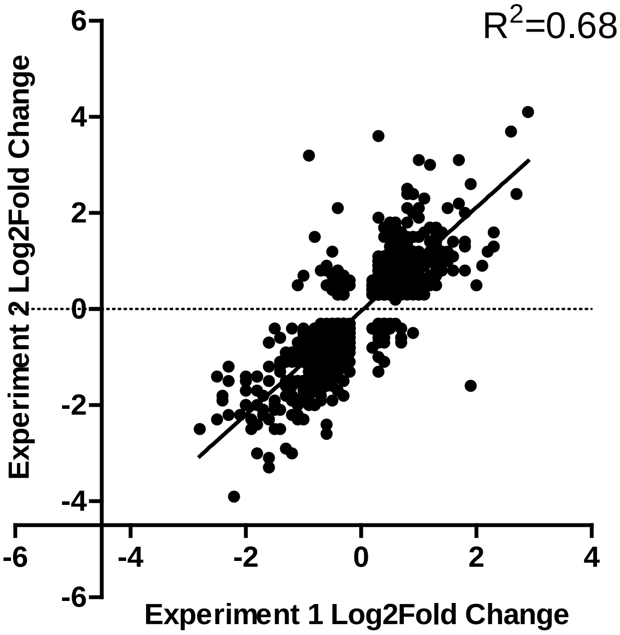

***Figure S2*: Conservation of cholesterol induced changes in macrophage gene expression in two independent experiments.** Linear regression analysis of log2 fold changes of the 1,140 overlapping transcripts between experiment 1and experiment 2 dataset that are significantly regulated by cholesterol loading in one or both strains. P-value of linear regression <0.0001.
